# Socioeconomic determinants of the leprosy risk in Yunnan Province, China: a county-level spatiotemporal study

**DOI:** 10.3389/fpubh.2025.1427319

**Published:** 2025-04-30

**Authors:** Jian Qian, Yue Ma, Yuxin Wei, Zutong Peng, Wei Li, Tao Zhang, Fei Yin, Tiejun Shui

**Affiliations:** ^1^West China School of Public Health and West China Fourth Hospital, Sichuan University, Chengdu, China; ^2^Department of Dermatology, the First Affiliated Hospital of Kunming Medical University, Yunnan, China; ^3^Yunnan Provincial Hospital of Traditional Chinese Medicine, Kunming, China; ^4^Yunnan Center for Disease Control and Prevention, Kunming, China

**Keywords:** leprosy, spatiotemporal study, socioeconomic factors, disease mapping, Bayesian hierarchical spatiotemporal model

## Abstract

**Background:**

Making leprosy history in low-burden countries is a crucial step in achieving the World Health Organization’s 2021–2030 Global Leprosy Strategy. Since leprosy has been contained at the national level in these countries, current efforts to eliminate leprosy have focused on specific leprosy high-risk areas. Quantifying the factors associated with leprosy risk would assist in identifying high-risk areas and the required efforts for leprosy elimination, which are still inadequate in these countries. To further progress a leprosy-free world, we investigated the associations between socioeconomic factors and the risk of leprosy in Yunnan Province, China.

**Methods:**

Socioeconomic factors and leprosy cases for 129 counties in Yunnan Province from 2004 to 2019 were collected. A spatiotemporal Bayesian model was used to evaluate the socioeconomic factors associated with leprosy risk.

**Results:**

4,361 leprosy cases were reported from 2004 to 2019 in Yunnan. Negative associations between disposable income per capita of rural residents (RR = 0.947, 95% CI: 0.907, 0.989), population density (RR = 0.920, 95% CI: 0.894, 0.945), the number of students enrolled by regular secondary schools (RR = 0.990, 95% CI: 0.986, 0.994), and leprosy risk were found in Yunnan Province. The associations between the per capita product of the primary industry, proportion of male population, number of beds in health and medical institutions per 1,000 population, and leprosy risk were not significant.

**Conclusion:**

This study revealed the associations between socioeconomic factors and leprosy risk in a low-burden country. These findings suggest that subsequent leprosy elimination efforts in Yunnan Province should prioritize rural areas, particularly those with lower population density and lower economic levels among farmers. Additionally, it is crucial to actively target poor rural farmers as a high-risk group for leprosy through strengthened early detection, multidrug therapy, and health education.

## Introduction

Leprosy, commonly known as Hansen’s disease (HD), is an infectious illness that has caused a heavy global disease burden during the past few decades (1). Caused by infection with *Mycobacterium leprae* and M lepromatosis, HD primarily impacts the peripheral nerves and skin, leading to lifelong damage and physical disabilities (2). Since the introduction of multidrug therapy (MDT), significant advancements have been achieved in the global leprosy elimination process, with the number of cases decreasing from over 5.4 million to 174,087 cases during the past decades (3). A major task in achieving the World Health Organization (WHO) 2021–2030 Global Leprosy Strategy “Towards Zero Leprosy” is to make leprosy history in low-burden countries (4). Since leprosy has been eliminated at the national level (prevalence < 1/100000), elimination efforts in these countries have primarily focused on the remaining endemic areas where leprosy cases still exist and other high-risk areas (5). Particularly considering the prolonged incubation time of leprosy and the pseudosilent areas of leprosy found in previous studies, the sustainable implementation of effective surveillance in high-risk areas is crucial to preventing the recurrence of leprosy (6). Therefore, identifying high-risk areas for leprosy is a critical step for these countries to achieve a leprosy-free world. To identify these high-risk areas, an effective approach is to analyze factors that are associated with leprosy risk. Given that poverty is strongly associated with leprosy, quantifying the socioeconomic factors related to leprosy risk enables more effective and efficient elimination efforts to be focused on these high-risk areas (7–9).

Several studies have revealed the associations between socioeconomic factors and leprosy in recent years (10–12). These studies were mainly carried out in countries with a high prevalence of leprosy, such as Brazil and India. For instance, the 100 million cohort study conducted in Brazil has shown that lower levels of income and education are associated with a significant increase in leprosy incidence. Specifically, the incidence rate ratio for the lowest versus highest quartile of income per capita was 1.46 (95% CI: 1.32–1.62), and for the lowest versus highest level of education, it was 2.09 (95% CI: 1.62–2.72) (13). Another study conducted in India reported only weak evidence of an association between poverty and leprosy case detection rates at the district level, although potential under-detection in impoverished areas may have biased the findings (14). However, few studies have quantified the socioeconomic factors associated with leprosy risk in low-burden countries, resulting in an inadequate understanding of high-risk areas requiring targeted leprosy elimination efforts within these countries. To further progress leprosy elimination worldwide, a deeper understanding of the socioeconomic factors associated with leprosy is needed in these countries.

Considering the spatial distribution of leprosy is typically uneven, ignoring spatial effects when investigating the factors associated with leprosy may lead to inaccurate or even erroneous results (15–17). Therefore, spatial analysis techniques such as Bayesian spatial models and spatial lag models have been employed to explore the influencing factors of leprosy (8, 18, 19). In addition to spatial effects, the temporal trend of leprosy should also be taken into account with a distinct decreasing pattern (20–22). Two studies included calendar time as a fixed effect when investigating socioeconomic factors related to leprosy detection rates using linear mixed-effects models but without considering spatial effects (14, 23). However, to our knowledge, no study has quantified the associations between socioeconomic factors and leprosy with a spatiotemporal model that adequately considers the potential spatiotemporal effects of leprosy.

With 136 newly discovered leprosy cases in 2019, Yunnan Province has the highest number of leprosy cases in China, exceeding cases reported by the majority of countries around the world (24). To assist policymakers in further implementing targeted strategies for leprosy elimination in Yunnan Province and provide guidance for leprosy elimination in low-burden countries worldwide, we carried out spatiotemporal research to quantify the associations between socioeconomic factors and leprosy. Specifically, we employed a Bayesian spatiotemporal model to analyze the associations between socioeconomic factors and leprosy in 129 counties in Yunnan Province from 2004 to 2019 based on the spatiotemporal correlation of leprosy found in a previous study (25).

## Materials and methods

### Study region

Located between latitudes 21°8’N and 29°15’N and longitudes 97°31′E and 106°11′E, Yunnan is an inland province on the southwestern border of China with an area of 394,100 square kilometers. Yunnan Province, with a total population of 48.58 million (2019), is divided into 16 prefecture-level cities and 129 counties, including 29 autonomous counties. Yunnan Province, with the highest leprosy burden, is also distinguished as the region with the greatest ethnic diversity in China.

### Data sources

Daily reported leprosy cases from January 1, 2004, to December 31, 2019 in Yunnan Province were obtained from the Leprosy Management Information System database in China. All cases were diagnosed based on clinical, bacteriological, and histopathological profiles, in according with the Diagnostic Standards for Leprosy, issued by the Health Industry Standard of the People’s Republic of China.^1^

The county-level annual socioeconomic factors during the study period were collected from the Yunnan Statistical Yearbook^2^ and the China Statistical Yearbook (County-Level).^3^ Based on previous research findings, 14 factors from four aspects were collected in the study. Economic factors included per capita gross domestic product (PCGDP), per capita product of the primary industry (PCPPI), per capita product of the secondary industry (PCPSI), per capita product of the tertiary industry (PCPTI), disposable income per capita of rural resident (DIPCRR), per capita savings deposits of rural and urban residents (PCSDR), per capita public budgetary revenue of local government (PCPBR), per capita public budgetary government expenditure of local government (PCPBGE), and per capita added value of primary industry (PCAVPI). Demographic factors included population density (PD) and proportion of male population (PMP). Education factors included the number of students enrolled by regular secondary schools (NSERSS) and the number of students enrolled by primary schools (NSEPS). Health factor included the number of beds in health and medical institutions per 1,000 population (NBHMI).

Since four factors (PCAVPI, NSERSS, NSEPS, and NBHMI) collected from the China Statistical Yearbook were only available for 120 counties, the missing data for the remaining 9 counties was estimated by using the average values from adjacent counties. The missing values (<1%) of the DIPCRR and PCSDR were imputed by linear interpolation.

### Statistical analysis

A spatiotemporal Bayesian model was employed to quantify the socioeconomic factors associated with leprosy risk. Considering the overdispersion of leprosy cases, a negative binomial distribution family was chosen in the model:


(1) $$Y_{\mathrm{it}}\sim \mathrm{NegBin}\left(E_{\mathrm{it}}\cdot \rho_{\mathrm{it}},\kappa \right)$$

(2) $$\log\left(\rho_{\mathrm{it}}\right)=\alpha +\sum \beta X_{\mathrm{it}}+\upsilon_{\mathrm{it}}+\nu_{\mathrm{it}}+\gamma_{t}$$

where $Y_{\mathrm{it}}$ is the leprosy cases for county i ( i =1, 2, …,129) in year t ( t =1, 2, …, 16); $E_{\mathrm{it}}$ and $\rho_{\mathrm{it}}$ are the expected number of leprosy cases and the relative risk (RR) of leprosy, respectively (Equation 1); and $X_{\mathrm{it}}$ is the socioeconomic factors included in the model (Equation 2). To account for the spatial random effects, the Besag-York-Mollie model is specified, which incorporates a year-specific conditional autoregressive model ( $\upsilon_{\mathrm{it}}$ ) and exchangeable random effect ( $\nu_{\mathrm{it}}$ ) (26). The first-order random walk prior was used to consider the temporally structured effect $\gamma_{t}$ . The variance inflation factor (VIF) and Spearman’s correlation coefficient were used to assess the correlation between these socioeconomic factors, which avoided the impact of multicollinearity that arises when highly correlated variables are simultaneously included in the model.

The parameters in the model were estimated with Integrated Nested Laplace Approximation (27). The model’s goodness of fit was evaluated by the deviance information criterion (DIC), where a smaller number denotes a better fit (28). Sensitivity analyses were conducted by specifying several minimally informative priors for the precision parameters of the spatial and temporal random effects to evaluate the robustness of the model estimates. All statistical analyses conducted in the study were implemented using R (version 4.1.3) with the packages *INLA*, *spdep*, and *rgdal*.

## Results

**Figure 1:**
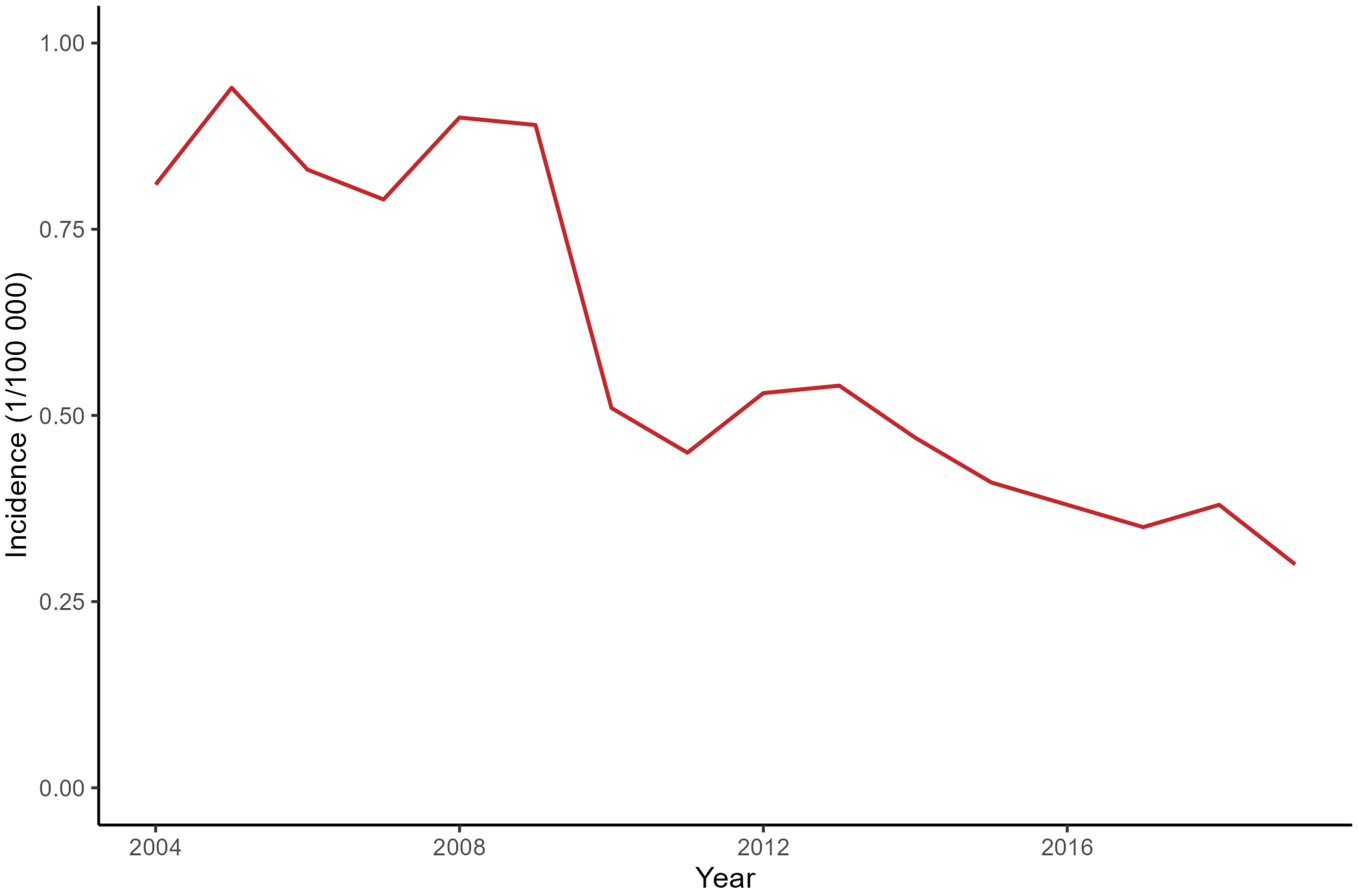
Annual incidence of leprosy in Yunnan Province from 2004 to 2019.

**Figure 2:**
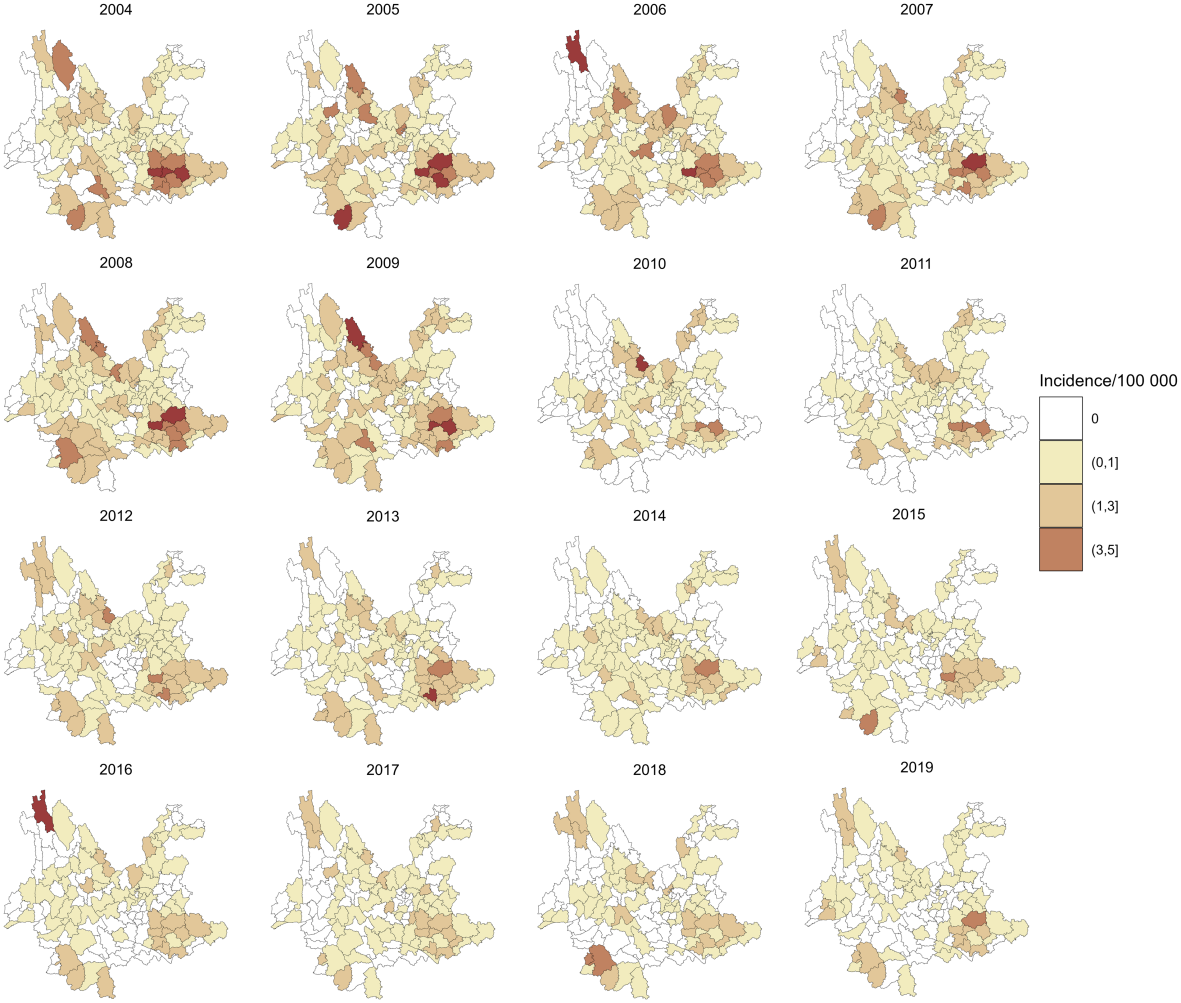
Annual incidence of leprosy in 129 counties in Yunnan Province from 2004 to 2019.

A total of 4,361 leprosy cases were detected in 129 counties of Yunnan Province from January 1, 2004, to December 31, 2019. Figure 1 shows the annual incidence of leprosy in Yunnan Province from 2004 to 2019, which exhibits a clear downward trend. Table 1 provides the descriptions of leprosy cases and socioeconomic factors in this study. The annual leprosy cases among the 129 counties ranged from 0 to 31, among which Kaiyuan City had the highest leprosy cases in 2005. Additionally, 8 counties had a total of more than 100 leprosy cases over the entire study period (2004–2019), with Qiubei County reporting the highest total number of cases at 251. Figure 2 illustrates the annual incidence of leprosy (1/100,000) among the 129 counties from 2004 to 2019. Overall, the leprosy incidence shows a downward trend and is primarily occurring in the southwestern, southeastern, and northern regions. During the study period, the highest leprosy incidence occurred in Kaiyuan City in 2005, with an incidence of 9.81/100000. But there were only 14 counties with an incidence exceeding 1/100,000 in 2019, and the highest incidence was 3.21/100000.

**Table 1:** Descriptions of leprosy cases and socioeconomic factors in 129 counties from 2004 to 2019.

Variables	Mean (SD)	*P*_1_	*P*_25_	Median	*P*_75_	*P*_99_	Range
Leprosy (cases)	2.11 (3.41)	0.00	0.00	1.00	3.00	17.00	(0.00, 31.00)
PCGDP (1000CNY)	20.02 (19.15)	2.31	7.58	14.74	25.25	106.15	(1.40, 163.08)
PCPPI (1000CNY)	3.62 (2.34)	0.28	1.83	3.04	4.89	10.64	(0.12, 14.69)
PCPSI (1000CNY)	7.95 (10.08)	0.42	2.36	5.05	9.42	52.29	(0.27, 98.53)
PCPTI (1000CNY)	8.37 (9.99)	0.78	2.54	5.05	10.05	52.13	(0.47, 95.42)
PD (100 people per km^2^)	1.92 (3.14)	0.09	0.78	1.22	1.87	21.75	(0.08, 28.20)
PMP (%)	51.79 (1.02)	49.79	51.06	51.74	52.48	54.06	(48.47, 55.36)
DIPCRR (1000CNY)	6.02 (4.09)	1.00	2.55	4.90	8.88	17.64	(0.70, 22.37)
PCSDR (10000CNY)	1.45 (1.77)	0.10	0.47	1.00	1.83	7.29	(0.06, 28.28)
PCPBR (1000CNY)	1.25 (1.19)	0.07	0.41	0.93	1.66	5.33	(0.04, 12.24)
PCPBGE (1000CNY)	5.28 (4.56)	0.60	1.88	4.59	7.39	22.74	(0.35, 53.87)
PCAVPI (100 million CNY)	12.55 (10.12)	0.93	5.41	9.58	16.82	47.72	(0.01, 73.06)
NSERSS (1,000 persons)	19.05 (15.50)	1.76	10.03	15.38	21.82	101.34	(1.29, 125.62)
NSEPS (1,000 persons)	30.69 (25.08)	3.78	15.92	24.16	37.31	148.20	(2.72, 243.01)
NBHMI (beds per 1,000 persons)	3.31 (2.05)	0.70	1.85	2.85	4.24	11.34	(0.53, 16.24)

P1, p25, p75, and p99 represent the 1st, 25th, 75th, and 99th percentiles of each variable, respectively.

**Figure 3:**
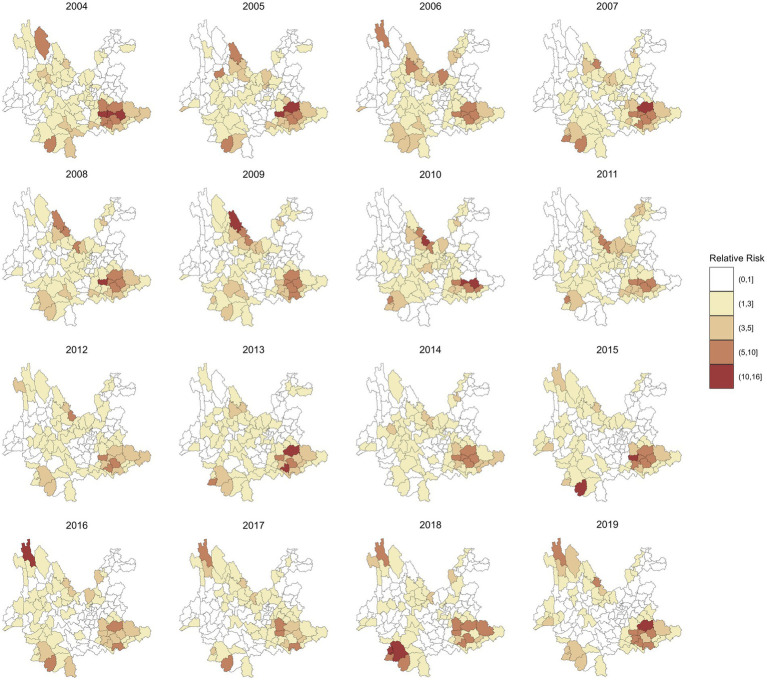
RR of leprosy in 129 counties from 2004 to 2019 by the spatiotemporal model without covariables.

Figure 3 shows the annual RR of leprosy in 129 counties in Yunnan Province from 2004 to 2019, which was estimated by the spatiotemporal model without covariates included. Compared to the annual incidence of leprosy in Figure 2, the RR values exhibit a smoother spatial distribution. The counties with the highest RR of leprosy were Ximeng Va Nationality Autonomous County in 2018 (RR: 15.92, 95% CI: 5.19, 39.16) and Kaiyuan City in 2005 (RR: 15.29, 95% CI: 9.07, 24.34). In 2019, the 95% CI of the RR exceeded 1 in only 13 counties, namely, Qiubei County, Dêqên County, Pingbian Miao Autonomous County, Huaping County, Mengzi City, Yanshan County, Kaiyuan City, Maguan County, Menghai County, Mang City, Lancang Lahu Autonomous County, Guangnan County, and Wenshan City.

Spearman’s correlation coefficients among the 14 socioeconomic factors are presented in Supplementary Table 1. The results indicate a high correlation (*r* > 0.8) among the seven economic factors: PCGDP, PCPSI, PCPTI, DIPCRR, PCSDR, PCPBR, and PCPBGE. Given that most leprosy cases in this study are farmers, only disposable income per capita of rural resident (DIPCRR) was included in the model to avoid multicollinearity. In addition, the number of students enrolled by regular secondary schools and primary schools are also highly correlated (*r* = 0.94). Considering that the number of students in regular secondary school reflects the local education level better than the number of students in primary school, the number of students enrolled by primary schools was not included in the model. Therefore, six variables were included in the final model: disposable income per capita of rural resident, per capita product of the primary industry, population density, proportion of male population, the number of students enrolled by regular secondary schools, and the number of beds in health and medical institutions per 1,000 population. The VIF of these variables (VIF < 4) indicate that there is no strong multicollinearity among them (Supplementary Table 2). Table 2 displays the DIC values for different models, indicating that the model incorporating spatiotemporal effects is the best-fitting model (DIC = 6668.943). The results of sensitivity analyses indicated robust model estimates across different prior specifications (Supplementary Table 3). Table 3 presents the coefficients and RR of the socioeconomic variables in the spatiotemporal model. These results indicate a negative correlation between disposable income per capita of rural resident, population density, the number of students enrolled by regular secondary schools, and leprosy. An increase of 1000 (CNY) in disposable income per capita of rural resident was associated with a 5.3% (95% CI: 1.1, 9.3%) decrease in the RR of leprosy. And increase of 100 (people per km^2^) in population density and 1,000 in students enrolled by regular secondary schools was associated with 8% (95% CI: 5.5, 10.6%) and 1.1% (95% CI: 0.6, 1.4%) decrease in the RR of leprosy, respectively. However, no significant association was observed between per capita product of the primary industry, proportion of male population, the number of beds in health and medical institutions per 1,000 population, and leprosy in this study. Supplementary Figures 1–6 illustrate the annual spatial distribution of the six socioeconomic variables above, it is evident that areas with lower disposable income per capita of rural resident (DIPCRR), lower population density (PD), and fewer students enrolled by regular secondary schools (NSERSS) tend to have higher incidence of leprosy.

**Table 2:** The DIC values of models with/without spatiotemporal effects.

Model	Specification	DIC
Base model	$\log\left(\rho_{\mathrm{it}}\right)=\alpha +\sum \beta X_{\mathrm{it}}$	7,527.878
Spatial model	$\log\left(\rho_{\mathrm{it}}\right)=\alpha +\sum \beta X_{\mathrm{it}}+\upsilon_{\mathrm{it}}+\nu_{\mathrm{it}}$	7,005.398
Spatiotemporal model	$\log\left(\rho_{\mathrm{it}}\right)=\alpha +\sum \beta X_{\mathrm{it}}+\upsilon_{\mathrm{it}}+\nu_{\mathrm{it}}+\gamma_{t}$	6,668.943

**Table 3:** The coefficients (*β*) and RR of the spatiotemporal model with six socioeconomic variables.

Variable	*β* (95% CI)	RR (95% CI)
DIPCRR	−0.054 (−0.097, −0.011)	0.947 (0.907, 0.989)
PCPPI	−0.015 (−0.055, 0.025)	0.985 (0.946, 1.026)
PD	−0.084 (−0.112, −0.057)	0.920 (0.894, 0.945)
PMP	−0.042 (−0.100, 0.016)	0.959 (0.905, 1.016)
NSERSS	−0.010 (−0.015, −0.006)	0.990 (0.986, 0.994)
NBHMI	0.024 (−0.006, 0.053)	1.024 (0.994, 1.055)

**Figure 4:**
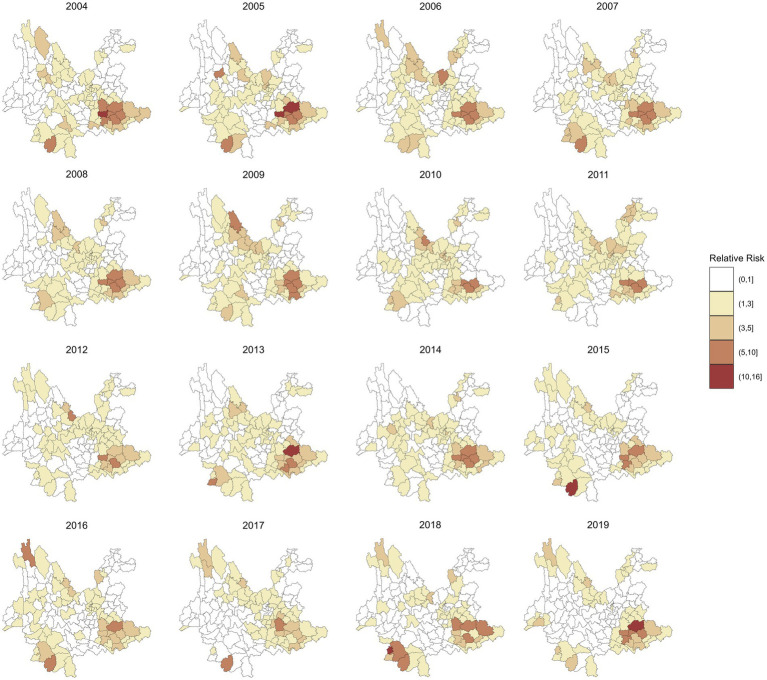
RR of leprosy in 129 counties from 2004 to 2019 by the multivariate spatiotemporal model.

Figure 4 maps the spatial distributions of the annual RR of leprosy in 129 counties in Yunnan Province from 2004 to 2019, which were estimated by a multivariate spatiotemporal model. Compared with the RR in Figure 3, these findings show that the inclusion of socioeconomic variables has reduced the heterogeneity in the spatial distribution of leprosy RR. For example, the highest RR of leprosy in Ximeng Va Nationality Autonomous County in 2018 decreased from 15.92 (95% CI: 5.19, 39.16) to 10.59 (95% CI: 3.49, 26.15) after the socioeconomic variables were included. The highest RR of leprosy in Figure 4 is 13.40 (95% CI: 7.94, 21.59) in Kaiyuan City in 2005.

## Discussion

With the implementation of the multifaceted leprosy elimination strategy in recent decades, the reported leprosy cases in Yunnan Province have decreased significantly. The upcoming leprosy elimination strategy in Yunnan Province will prioritize the remaining leprosy-endemic counties and the prevention of leprosy recurrence, aligning with the target of the WHO Global Leprosy Strategy 2021–2030, aiming to achieve a leprosy-free world. In this study, we conducted a spatiotemporal analysis in 129 counties in Yunnan Province to uncover the associations between socioeconomic factors and leprosy risk. This is the first spatiotemporal study quantifying the association between socioeconomic factors and leprosy in leprosy low-burden country. These findings indicate that the targeted strategy for leprosy elimination in Yunnan Province should prioritize rural areas with lower levels of economic and educational attainment and also provide a reference for leprosy elimination efforts in other low-burden countries worldwide.

Leprosy is often considered a disease driven primarily by poverty, and lower economic conditions are commonly linked to a higher leprosy risk. The associations between two economic factors and leprosy risk were analyzed in this study. The results indicate that rural residents with higher disposable income or savings have a lower risk of leprosy. This suggests that efforts to eliminate leprosy should be prioritized in areas where farmers have lower economic levels. This finding aligns with previous research conducted in other countries, consistently indicating poverty is a significant driver of risk to those coming down with leprosy (13, 14, 20, 29, 30). This evidence also indicates that the poor living conditions associated with poverty may increase the risk of leprosy, while the physical disabilities and discrimination caused by leprosy can delay treatment and contribute to further economic decline (31). As a result, a bidirectional association between poverty and leprosy has been formed. Therefore, in rural areas with lower income levels, early diagnosis and treatment should be improved to prevent disability, and more social support should be provided for leprosy patients to eliminate the discrimination caused by leprosy. In addition, the association between the per capita product of the primary industry (PCPPI) and the risk of leprosy was not significant. The potential reason could be that most of the newly reported leprosy cases in Yunnan Province are farmers (>90%), while the PCPPI is calculated based on the total population, ignoring the occupational composition within the population (32).

Regarding demographic factors, the associations between population density, the proportion of male population, and leprosy risk were quantified in this study. The results revealed a negative association between population density and leprosy risk, which is inconsistent with the results observed in other high-burden leprosy countries. For example, studies in Brazil have shown that areas with higher urbanization rates or a higher proportion of urban populations are associated with a higher risk of leprosy (8, 13, 33). These inconsistent associations could be attributed to the prevalence of leprosy in the inaccessible mountainous areas of Yunnan Province, which are primarily located in sparsely populated rural areas (34). The discovery suggests significant advancements in the leprosy elimination strategy implemented in Yunnan Province during the past decades, emphasizing the need for further promotion of multidrug therapy and early detection in areas with low population density. Moreover, these discrepancies also indicate that leprosy elimination efforts in low-burden countries may need to be focused on sparsely populated areas rather than urban areas in high-burden countries. In addition, these studies conducted in high-burden countries also showed a positive association between household density (average individuals per room) and leprosy (13, 33, 35). Considering that roughly 30% of newly detected leprosy cases in Yunnan Province have a history of family contact, early case detection and contact surveillance for new leprosy cases should be further strengthened to reduce intrafamilial transmission of leprosy (36). The relationship between the proportion of male population and leprosy was not significant in this study. Although the proportion of males among newly reported leprosy cases in Yunnan Province from 2004 to 2019 was significantly higher than that of females (male-to-female ratio = 2.39, *p* < 0.01), a meta-analysis also indicated that males had a higher leprosy risk (RR = 1.33, 95% CI: 1.06, 1.67) (36, 37). This insignificant association may be attributed to the relatively small variation in male-to-female ratios at the county level in Yunnan Province. For instance, among the 2064 units analyzed in this study, the interquartile range of the proportion of male population was 51.06 to 52.48%.

For the education factor, a negative association was found between the number of students enrolled by regular secondary schools and leprosy risk. According to the inequality of secondary education, secondary school students are more likely to be concentrated in urban areas (38). This suggests that the population in these areas may be more educated, have greater health awareness, and engage in healthier behaviors, thereby reducing the risk of leprosy infection through early detection. Therefore, leprosy health education should be further strengthened in areas with low educational attainment, particularly targeting the rural population, to reduce transmission. The positive association between illiteracy/lower education and leprosy has also been confirmed in several studies (13, 30, 33, 39, 40). Regarding health factor, the number of beds in health and medical institutions per 1,000 population was not associated with leprosy in this study. This result may suggest that it is not the absolute level of medical resources that limits the progress of leprosy elimination but the specific elimination efforts, including early detection and MDT. Inconsistent associations between different metrics of healthcare services and leprosy were also observed in previous studies. For instance, the number of health care facilities provided by the Program of Leprosy and coverage of Family Health Program were found to be positively correlated with leprosy in Brazil (30, 33, 41). However, there were no significant associations presented between the number of physicians per 1,000 population, number of public health services per 1,000 population, vaccination coverage, and leprosy (40, 42). Although these results suggest that socioeconomic factors partly explain the risk of leprosy, the remaining spatial heterogeneity of leprosy risk highlights the need to explore additional county-level factors. Further exploration is needed regarding metrics related to early leprosy detection and the accessibility of healthcare services in rural areas.

This study has several strengths. To our knowledge, it is the first spatiotemporal analysis to quantify the association between socioeconomic factors and leprosy in a leprosy low-burden country. By identifying high-risk areas and key socioeconomic determinants, our findings provide important evidence to guide targeted leprosy elimination strategies in Yunnan Province and offer valuable insights for other countries with similar epidemiological contexts. Despite these contributions, some limitations should be acknowledged. First, some factors that may be associated with the risk of leprosy, such as age, ethnic group, living conditions, and accessibility to healthcare services, were not included in this study due to limited data availability. Future research can be conducted to explore these associations when data become available. Second, only data from a 16-year period were analyzed in this study, and longer-term data would contribute to a more robust estimation of these associations. However, the establishment of a special fund for leprosy in 2004 and the outbreak of COVID-19 at the end of 2019 may have resulted in great heterogeneous associations between socioeconomic factors and leprosy risk. Therefore, the time scope was limited to 2004–2019 to obtain more stable association results.

## Conclusion

In conclusion, this study revealed a negative association between the disposable income per capita of rural residents, population density, the number of students enrolled by regular secondary schools, and the risk of leprosy in Yunnan Province. These findings highlight the differences in priority areas for leprosy elimination between low-and high-burden countries and suggest that subsequent leprosy elimination efforts in Yunnan Province should prioritize rural areas with lower population density and lower economic levels among farmers. Importantly, poor rural farmers should be actively targeted as a high-risk group, emphasizing the need for focused interventions to address their vulnerabilities.

## Data Availability

The datasets analyzed in this study are available from Yunnan Center for Disease Control and Prevention. The authors used the data for this current study under a license from the Yunnan CDC, so the data cannot be shared publicly. Requests to access these datasets should be directed to yncdcjsb@yncdc.cn.

## Footnotes

^1^ https://icdc.chinacdc.cn/zcfgybz/bz/202112/t20211202_253307.html

^2^ https://stats.yn.gov.cn/

^3^ https://www.stats.gov.cn/
