# Supplementary material for: Socioeconomic determinants of the leprosy risk in Yunnan Province, China: a county-level spatiotemporal study
Source: Front Public Health. 2025 Apr 30;13:1427319. doi: 10.3389/fpubh.2025.1427319 (PMC12075559; doi:10.3389/fpubh.2025.1427319)
Supplement: Supplementary file 1 [file Table_1.docx]

**Supplementary Material**

**Socioeconomic determinants of the leprosy risk in Yunnan Province, China: a county-level spatiotemporal study**

Jian Qian ^1^†, Yue Ma ^1^†, Yuxin Wei ^1^, Zutong Peng ^2^, Wei Li ^3^, Tao Zhang^1^, Fei Yin ^1*^, Tiejun Shui ^4*^

1: West China School of Public Health and West China Fourth Hospital, Sichuan University, Chengdu, China

2: Department of Dermatology, The First Affiliated Hospital of Kunming Medical University, Yunnan, China

3: Yunnan Provincial Hospital of Traditional Chinese Medicine, Kunming, China

4: Yunnan Center for Disease Control and Prevention, Kunming, China

†These authors contributed equally to this work and share first authorship.

*: Author for correspondence:

yinfeiscuedu@163.com (Fei Yin);

[shuitiejunynjkedu@163.com](mailto:shuitiejunynjkedu@163.com) (Tiejun Shui)

# 1 Supplementary Tables

**Supplementary Table 1. Spearman Correlation Coefficients Between socioeconomic variables of the 129 Counties in Yunnan Province.**

| Variable | PCGDP | PCPPI | PCPSI | PCPTI | PD | PMP | DIPCRR | PCSDR | PCPBR | PCPBGE | PCAVPI | NSERSS | NSEPS | NBHMI |
| --- | --- | --- | --- | --- | --- | --- | --- | --- | --- | --- | --- | --- | --- | --- |
| PCGDP | 1.00 | 0.58 | 0.95 | 0.96 | 0.23 | -0.03 | 0.89 | 0.93 | 0.93 | 0.72 | 0.58 | -0.02 | -0.15 | 0.61 |
| PCPPI | 0.58 | 1.00 | 0.45 | 0.56 | -0.14 | -0.03 | 0.71 | 0.54 | 0.56 | 0.69 | 0.68 | -0.07 | -0.16 | 0.61 |
| PCPSI | 0.95 | 0.45 | 1.00 | 0.86 | 0.26 | -0.01 | 0.78 | 0.85 | 0.90 | 0.62 | 0.51 | 0.05 | -0.08 | 0.55 |
| PCPTI | 0.96 | 0.56 | 0.86 | 1.00 | 0.22 | -0.04 | 0.88 | 0.94 | 0.90 | 0.73 | 0.54 | -0.06 | -0.19 | 0.62 |
| PD | 0.23 | -0.14 | 0.26 | 0.22 | 1.00 | -0.15 | 0.26 | 0.27 | 0.19 | -0.15 | 0.29 | 0.49 | 0.45 | 0.01 |
| PMP | -0.03 | -0.03 | -0.01 | -0.04 | -0.15 | 1.00 | 0.00 | -0.10 | -0.04 | 0.17 | -0.02 | 0.04 | 0.11 | -0.10 |
| DIPCRR | 0.89 | 0.71 | 0.78 | 0.88 | 0.26 | 0.00 | 1.00 | 0.88 | 0.84 | 0.80 | 0.71 | 0.05 | -0.08 | 0.62 |
| PCSDR | 0.93 | 0.54 | 0.85 | 0.94 | 0.27 | -0.10 | 0.88 | 1.00 | 0.91 | 0.72 | 0.52 | -0.04 | -0.18 | 0.66 |
| PCPBR | 0.93 | 0.56 | 0.90 | 0.90 | 0.19 | -0.04 | 0.84 | 0.91 | 1.00 | 0.73 | 0.52 | -0.06 | -0.20 | 0.64 |
| PCPBGE | 0.72 | 0.69 | 0.62 | 0.73 | -0.15 | 0.17 | 0.80 | 0.72 | 0.73 | 1.00 | 0.43 | -0.23 | -0.34 | 0.64 |
| PCAVPI | 0.58 | 0.68 | 0.51 | 0.54 | 0.29 | -0.02 | 0.71 | 0.52 | 0.52 | 0.43 | 1.00 | 0.54 | 0.45 | 0.44 |
| NSERSS | -0.02 | -0.07 | 0.05 | -0.06 | 0.49 | 0.04 | 0.05 | -0.04 | -0.06 | -0.23 | 0.54 | 1.00 | 0.94 | 0.00 |
| NSEPS | -0.15 | -0.16 | -0.08 | -0.19 | 0.45 | 0.11 | -0.08 | -0.18 | -0.20 | -0.34 | 0.45 | 0.94 | 1.00 | -0.12 |
| NBHMI | 0.61 | 0.61 | 0.55 | 0.62 | 0.01 | -0.10 | 0.62 | 0.66 | 0.64 | 0.64 | 0.44 | 0.00 | -0.12 | 1.00 |

**Abbreviations**: PCGDP, per capita gross domestic product; PCPPI, per capita product of the primary industry; PCPSI, per capita product of the secondary industry; PCPTI, per capita product of the tertiary industry; PD, population density; PMP, proportion of male population; DIPCRR, disposable income per capita of rural resident; PCSDR, per capita savings deposits of rural and urban residents; PCPBR, per capita public budgetary revenue of local government; PCPBGE, per capita public budgetary government expenditure of local government; PCAVPI, per capita added value of primary industry; NSERSS, number of students enrolled by regular secondary schools; NSEPS, number of students enrolled by primary schools; NBHMI, number of beds in health and medical institutions per 1 000 population

**Supplementary Table 2. The VIF values of socioeconomic factors in spatiotemporal model.**

|  | DIPCRR | PCPPI | PD | PMP | NSERSS | NBHMI |
| --- | --- | --- | --- | --- | --- | --- |
| VIF | 3.49 | 2.64 | 1.45 | 1.05 | 1.08 | 1.60 |

**Abbreviations**: DIPCRR, disposable income per capita of rural resident; PCPPI, per capita product of the primary industry; PD, population density; PMP, proportion of male population; NSERSS, number of students enrolled by regular secondary schools; NBHMI, number of beds in health and medical institutions per 1 000 population.

**Supplementary Table 3. The RR of six socioeconomic variables across different priors.**

| Parameter | Prior*^a^* | DIPCRR | PCPPI | PD | PMP | NSERSS | NBHMI |
| --- | --- | --- | --- | --- | --- | --- | --- |
| $\tau$ | Gamma (1,0.0005) | 0.947  (0.907, 0.989) | 0.985  (0.946, 1.026) | 0.920  (0.894, 0.945) | 0.959  (0.905, 1.016) | 0.990  (0.986, 0.994) | 1.024  (0.994, 1.055) |
| $\tau$ | Gamma (1,0.0005) |  |  |  |  |  |  |
| $\tau$ | Gamma (1,0.00005) |  |  |  |  |  |  |
| $\tau$ | Gamma (1,0.001) | 0.948  (0.908,0.989) | 0.985  (0.947,1.026) | 0.918  (0.893,0.944) | 0.963  (0.909,1.020) | 0.988  (0.984,0.993) | 1.022  (0.993,1.053) |
| $\tau$ | Gamma (1,0.001) |  |  |  |  |  |  |
| $\tau$ | Gamma (1,0.001) |  |  |  |  |  |  |
| $\tau$ | Gamma (1,0.01) | 0.948  (0.908,0.990) | 0.986  (0.947,1.026) | 0.919  (0.894,0.945) | 0.963  (0.908,1.020) | 0.988  (0.984,0.993) | 1.023  (0.994,1.054) |
| $\tau$ | Gamma (1,0.01) |  |  |  |  |  |  |
| $\tau$ | Gamma (1,0.01) |  |  |  |  |  |  |
| $\tau$ | Gamma (1,0.1) | 0.947  (0.906,0.991) | 0.986  (0.947,1.027) | 0.920  (0.895,0.946) | 0.963  (0.909,1.021) | 0.988  (0.984,0.993) | 1.027  (0.996,1.058) |
| $\tau$ | Gamma (1,0.1) |  |  |  |  |  |  |
| $\tau$ | Gamma (1,0.1) |  |  |  |  |  |  |

*^a^*: The default prior distributions for $\tau$, $\tau$, and $\tau$ were Gamma (1,0.0005), Gamma (1,0.0005), and Gamma (1,0.00005), respectively.

**2 Supplementary Figures**

**
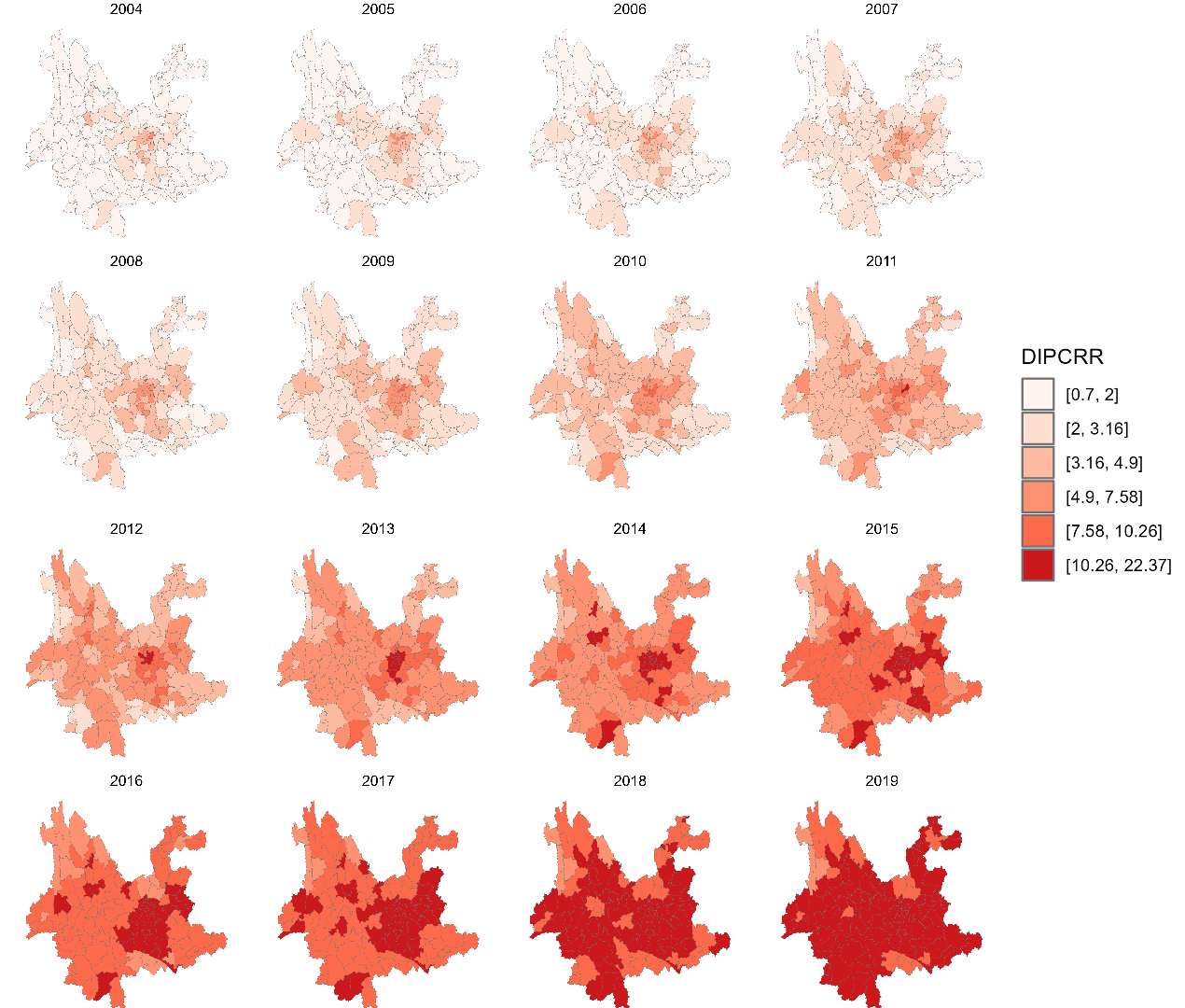
**

**Supplementary Figure 1 Annual county-level DIPCRR from 2004 to 2019 in Yunnan**

**
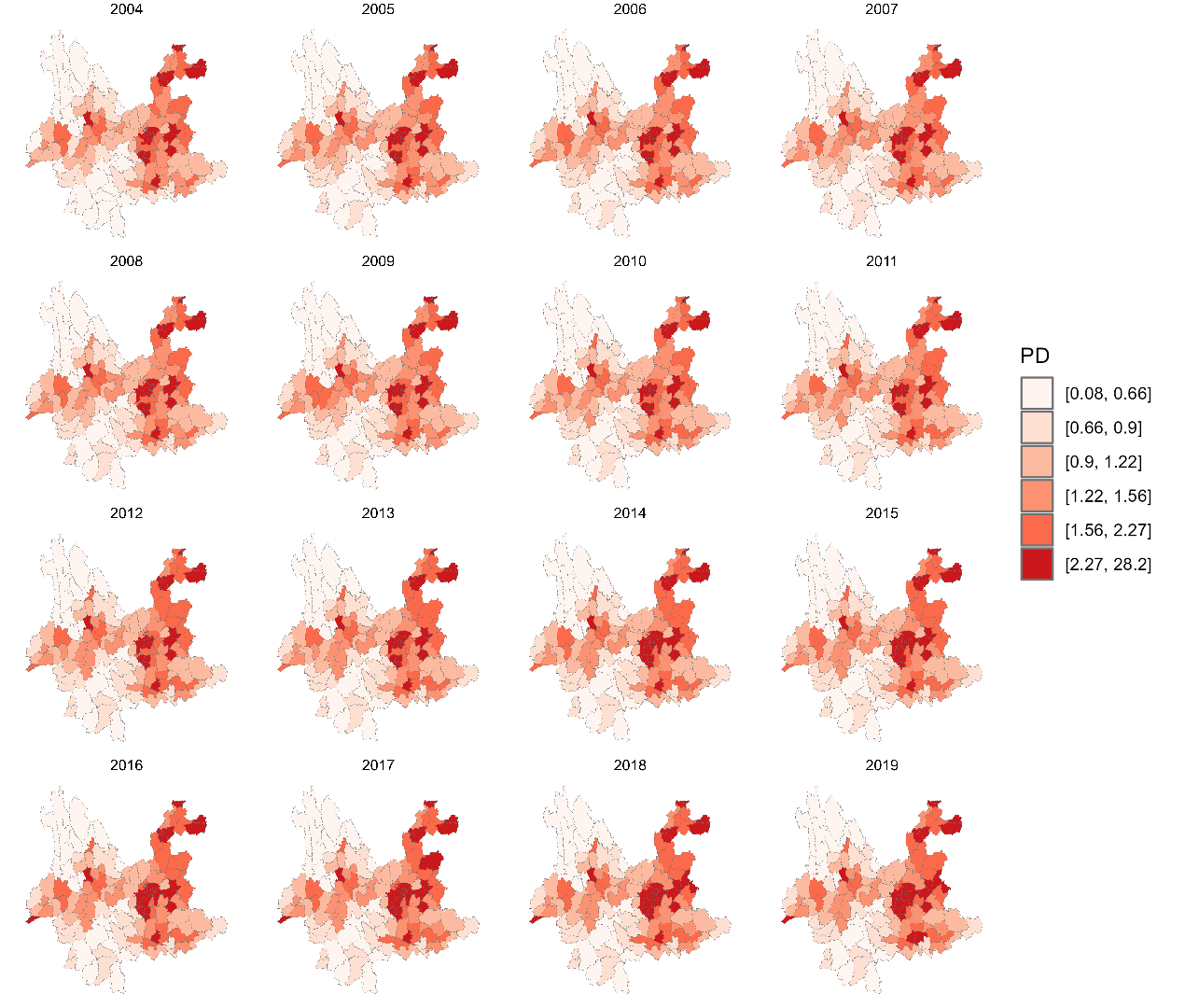
**

**Supplementary Figure 2 Annual county-level PD from 2004 to 2019 in Yunnan**

**
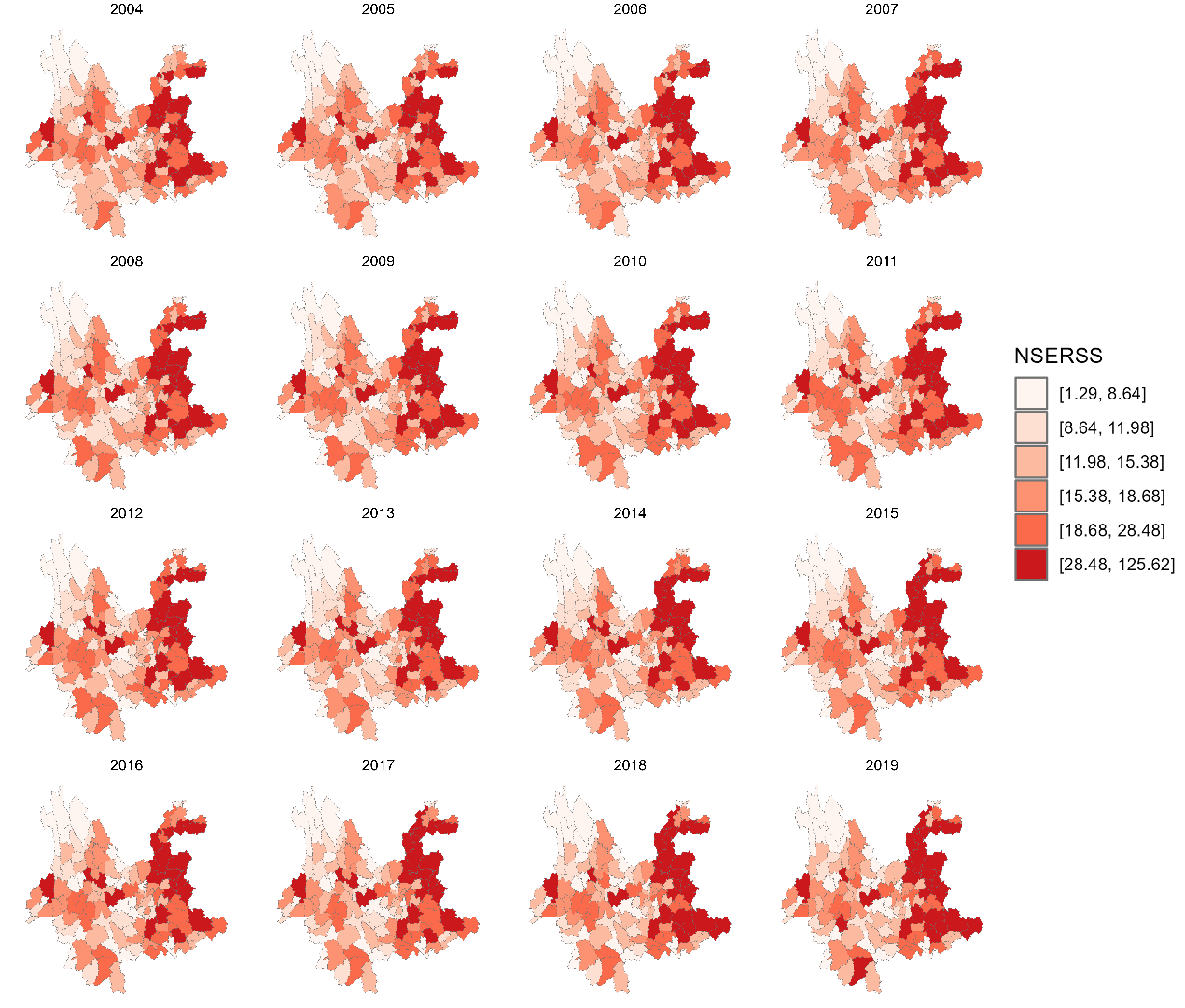
**

**Supplementary Figure 3 Annual county-level NSERSS from 2004 to 2019 in Yunnan**

**
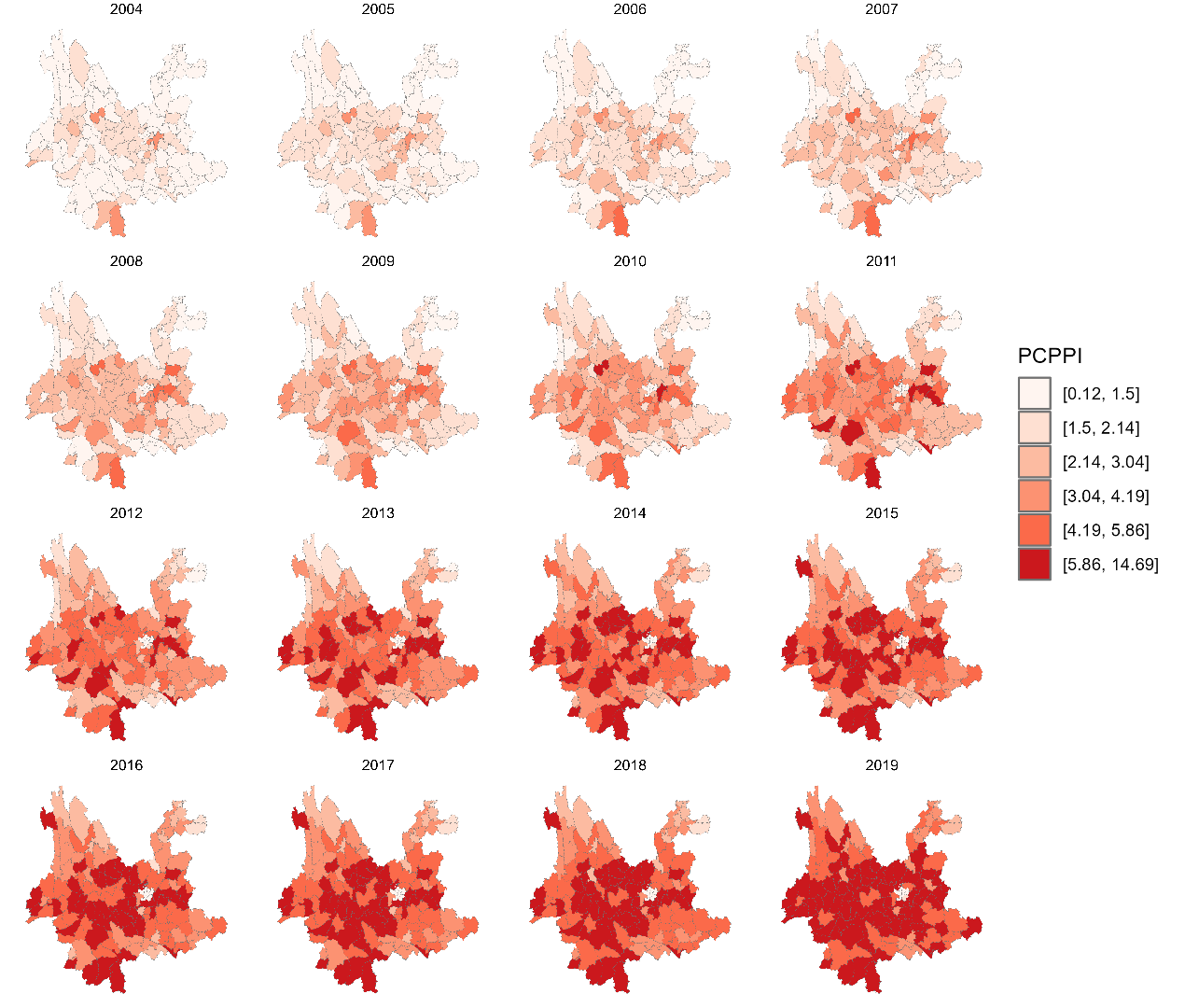
**

**Supplementary Figure 4 Annual county-level PCPPI from 2004 to 2019 in Yunnan**

**
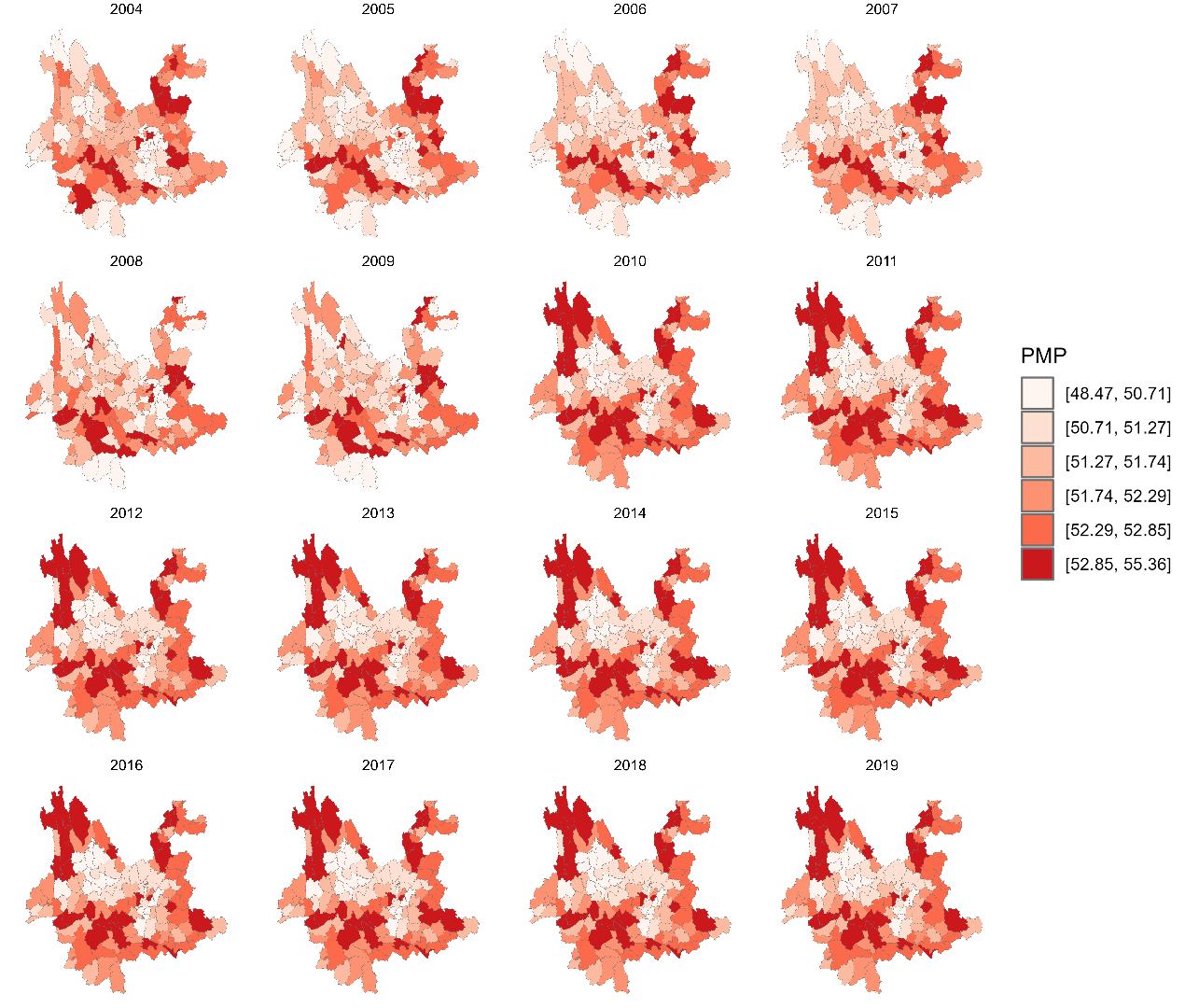
**

**Supplementary Figure 5 Annual county-level PMP from 2004 to 2019 in Yunnan**

**
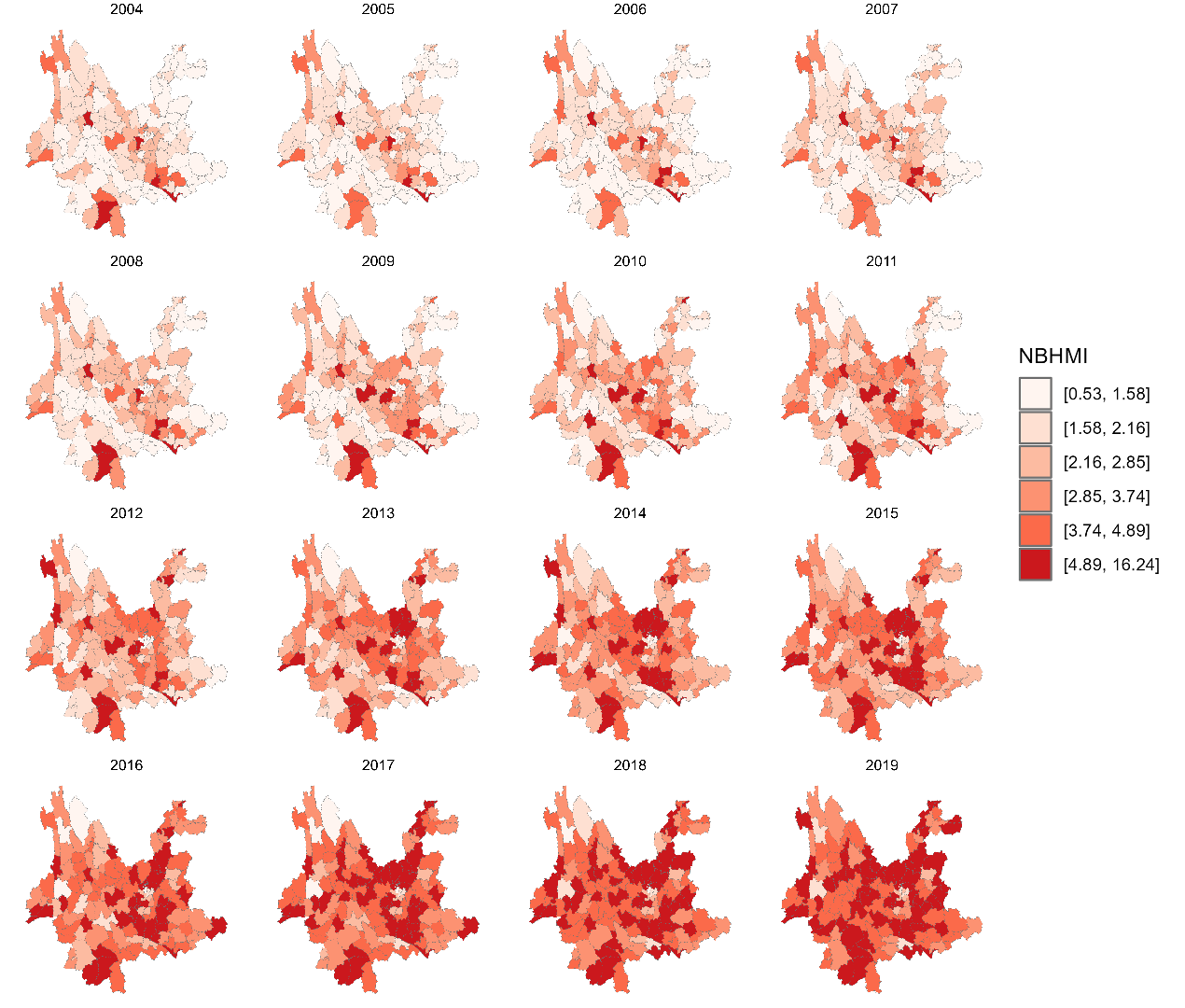
**

**Supplementary Figure 6 Annual county-level NBHMI from 2004 to 2019 in Yunnan**
